# Properties of the Vascular Networks in Malignant Tumors

**DOI:** 10.3390/e22020166

**Published:** 2020-01-31

**Authors:** Juan Carlos Chimal-Eguía, Erandi Castillo-Montiel, Ricardo T. Paez-Hernández

**Affiliations:** 1 Centro de Investigación en Computación del Instituto Politécnico Nacional, Av. Miguel Othon de Mendizabal s/n. Col. La Escalera, Ciudad de México CP 07738, Mexico; 2 Department of Técnologias WEB, Instituto Politécnico Nacional (IPN) - Centro Nacional de Cálculo (CENAC), Av. Luis Enrique Erro S/N, Unidad Profesional Adolfo López Mateos, Zacatenco, Gustavo A. Madero, Ciudad de México CP 07738, Mexico; erandicm@gmail.com; 3 Área de Física de Procesos Irreversibles, Departamento de Ciencias Básicas, Universidad Autónoma Metropolitana, U-Azcapotzalco, Av. San Pablo 180, Col.Reynosa, Ciudad de México CP 02200, Mexico; phrt@correo.azc.uam.mx

**Keywords:** complex networks, angiogenesis, network properties

## Abstract

This work presents an analysis for real and synthetic angiogenic networks using a tomography image that obtains a portrait of a vascular network. After the image conversion into a binary format it is possible to measure various network properties, which includes the average path length, the clustering coefficient, the degree distribution and the fractal dimension. When comparing the observed properties with that produced by the Invasion Percolation algorithm (IPA), we observe that there exist differences between the properties obtained by the real and the synthetic networks produced by the IPA algorithm. Taking into account the former, a new algorithm which models the expansion of an angiogenic network through randomly heuristic rules is proposed. When comparing this new algorithm with the real networks it is observed that now both share some properties. Once creating synthetic networks, we prove the robustness of the network by subjecting the original angiogenic and the synthetic networks to the removal of the most connected nodes, and see to what extent the properties changed. Using this concept of robustness, in a very naive fashion it is possible to launch a hypothetical proposal for a therapeutic treatment based on the robustness of the network.

## 1. Introduction

The development of cancer has thereby concentrated on an approach which is center around the genetic events which allow cells to escape from growth control and become cancerous. However, even if cancer cells have been generated and can evolve to accumulate more mutations, these cancer cells might not be able to grow beyond a very small size.

One of the most important factors in this respect is the blood supply which provides cancer cells with oxygen, nutrients and necessities required for survival. When the growth of tumoral cells is high enough to consume all supplies in a specific organ or tissue, the tumor stops its growth in order to induce the generation of new blood supply to sustain its growth; this process is called angiogenesis.

Whether a new blood supply can be formed or not appears to be determined by the balance between angiogenesis inhibitors and promoters. When angiogenic cell lines emerge they can shift the balance away from inhibition and in favor of promotion. This induces blood vessels to grow towards the tumor and this process leads to the complete vascularization of the tumor, i.e., a vascular network [1].

Our understanding about the role of angiogenesis in the development of cancers has advanced significantly since the studies of Judah Folkman [2]. Many of these studies have been focused mainly on the understanding of the cells at molecular level. To fully understand the behaviour of an organism, organ or even a single cell, we need to fully comprehend the collective behaviour of the whole system. Recently, the analysis of the behaviours of the biological systems or their emergent properties, that are not apparent from the examination of only a few isolated interactions alone, have emerged as new insights in the study of the systems in biology.

For instance, the use of fractal geometry [3] can describe the pathological structure of tumors, and give us some insights into the mechanisms of tumor growth and angiogenesis, that complement those obtained by modern molecular methods. Another good example is the comparative analysis of the transcription gene regulatory networks of the E. Coli and S. Cervisae made by Santillan et al. [4], or the interesting work made by Abdollahi et al. [5]. In both articles, the authors noticed how the network properties of the gene networks revealed some interesting data from an evolutionary point of view.

These among many others [6,7,8,9], are good examples of how emergent properties could help us understand the behaviour of biological systems. In particular, the use of network theory is important because it allows the description of a network structure using graph concepts. Furthermore, the observed network topology gives clues about the evolution, structure, which helps us elucidate the dynamics of hundreds of interacting components [10,11,12,13].

In this work, we present an analysis of two angiogenic networks in patients with Hepato-Cellular Carcinoma (HCC). We used a tomography image (obtained from the National Institute of Nutrition of México INNSZ) in order to obtain the vascular network. After the conversion of the image into a binary skeletonized form, we measured some of the network properties; the performed measurements includes the average path length, the clustering coefficient, the degree distribution, and the fractal dimension.

The observed properties of the tumor vasculature as a whole closely correspond to those produced by a new algorithm of random growth process known as Angiogenesis Random Growth Algorithm (ARGA). ARGA models the expansion of an angiogenic network through randomly heuristic rules. We test the network robustness by subjecting the original angiogenic and the synthetic networks produced by ARGA to the removal of the most connected nodes and seeing to what extent the properties changed (in particular the clustering coefficient). Taking into account this robustness, we proposed a hypothetic therapeutic treatment based on the network robustness.

The paper is organized as follows: In Section 2 we present the analysis of the angiogenic network, after converting into a binary skeletonized form from a tomography image and then analyzed its network properties. Section 3 presents a robustness study of the real network compared with those processed by a new algorithm called ARGA. Finally, Section 4 gives some concluding remarks.

## 2. Analysis of the Angiogenic Network

Angiogenesis is an important natural process that takes into account the growth of new blood vessels that occur in the body, both in health and disease. Angiogenesis is now recognized as one of the critical events required for tumor progression [14], where cancerous growth is dependent on vascular induction and the development of a new vascular supply.

The idea of targeting angiogenesis to inhibit tumor growth was proposed more than three decades ago, and since then, several approaches to block or disrupt tumor angiogenesis have been explored. However, all of these have been focused on the understanding of the molecular behaviour, and only a few in other properties that are not apparent from the molecular point of view.

For instance, some authors have declared: “Angiogenesis in tumors leads to tumor vessels with multiple functional and structural abnormalities. Tumors consist of a chaotic, poorly organized vasculature, with tortuous, irregular shape, and leaky vessels that are often unable to support efficient blood flow and leading to an aberrant vascular system” [15,16]. Furthermore, is it possible to consider that the tumor angiogenesis leads to a poorly organized vasculature without another measurement that the observation in situ. In our opinion, the answer to this question should be supported by some structural analysis. In this context we proposed the following analysis in order to have more elements that show if the vasculature network created by the tumor has this chaotic image, or has some structural elements that are not apparent only from observation that make them in some sense efficient to their purposes.

### 2.1. Creating the Network from a Image Tomography

The first step in our analysis is to obtain the vascular network from a tomography image. In mathematical terms a network is represented by a graph, which is a pair of sets G=(P,E), where *P* is a set of nodes (or vertices or points) $P_{1},P_{2},\ldots ,P_{n}$ and *E* is a set of edges (or links or lines) that connect two elements of *P*. Graphs are usually represented as a set of dots, each corresponding to a node, two of these dots being joined by a line if the corresponding nodes are connected.

From the department of radiology of the National Institute of Medical Sciences “Salvador Zuviran” (INNSZ) of Mexico City, we obtained images used by the INNSZ in order to diagnose the development of the malignant tumors of four patients with Hepato-Cellular Carcinoma (HCC) (see Table 1).

The progress in the development of the disease of these patients in the INNSZ is made by a computerized tomogram. This tomograph is not only dedicated to diagnosis of the angiogenesis process, but also other cancerous diseases such as the detection of gliomas, etc. The images taken by the tomogram have a resolution of 960 by 1260 pixels. These images are stored in DICOM format, and using the MedimaIView software we can manipulated it to obtain BMP images with a resolution of 960 by 1240 pixels (8 bits for pixel). From the BMP image we obtain different images with different sizes. In our study we obtained four sizes 32 by 32 pixels, 64 by 64 pixels 128 by 128 pixels and 256 by 256 pixels.

**Figure 1:**
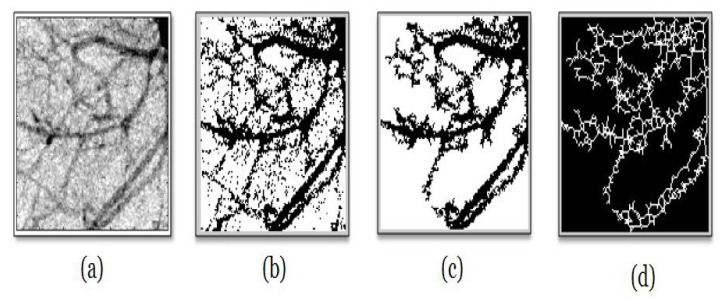
Step by Step of the digital processing make to the BMP image to obtain a binary skeletonized form: (**a**) image in gray scale, (**b**) Image in binary form, (**c**) Segmentation procedure, (**d**) Skeletonized binary form.

Once the image is obtained in a BMP format we proceed to make a digital processing of the image to obtain a binary skeletonized image. The procedure is as follows [17]:

1. Pre-Processing
  The tomographic images were subjected to a pre-processing stage to obtain the tumor vascular network. Using a representation of the image in 2-D, the first step was to display the image into a gray scale, where each pixel uses an individual value that represents its luminescence, and thus, have greater ease in handling the image. All the tomography images given by the INNSZ were very noisy, making it difficult to identify the blood vessels, so it was decided to make an improvement in the image by adjusting the contrast automatically.
  The representation of an image in an 2-D array is given by the intensity values f(x,y) at each image pixel. The arrangement has *M* rows and *N* columns, where (x,y) are discrete coordinates. We used for convenience integer values for discrete coordinates. Then we have for each coordinate x=0,1,2,…,M−1 and y=0,1,2,…,N−1. In a matrix representation obtaining
  (1) $$f\left(x,y\right)=\left[\begin{array}{cccc} f(0,0) & f(0,1) & \cdots & f(0,N-1)\\ \vdots & \vdots & \ddots & \vdots \\ f(M-1,0 & f(M-1,1) & \cdots & f(M-1,N-1) \end{array}\right]$$
  The gray scale adjustment consists of multiplying each RGB component by three constants defined by: α, β and γ. Subsequently, the intensity obtained in each channel is averaged.
  This process subtracts all the color information contained in each pixel and gives a separation of 255 levels between black and white.These three constants are obtained as the separation between the RGB and the black channels as:
  - α: Division between the red and black. (0.2989)
  - β: Division between the green and black. (0.5870)
  - γ: Division between the blue and black. (0.1140)
  Now to obtain the equivalent gray scale value for each pixel we use the following equation:
  I=α*R+β*G+γ*B
  We shall now proceed to the brightness adjustment as the last part of the stage of pre-processing algorithm. Brightness is the percentage of luminescence or darkness of a color. It is possible to go from 0 % which means black, up to 100% which means white. Mathematically, the operation corresponding to the brightness adjustment is: M+B=C, where M corresponds to the image matrix, C corresponds to the adjusted image M, and p is the parameter adjusting brightness whose standard ranges from −100 to 100.
2. Segmentation
  Now proceed to the image segmentation stage in which we obtain the angiogenic network by extracting most of the blood vessels that are connected within the image and store them in a new image. To achieve this we use a threshold which cleaves the image into two classes of objects: blood vessels and background image. Otsu’s method [18] calculates this threshold automatically in the following way: in order to find the value of a threshold *T*, for which the variance $\sigma_{B}^{2}\left(T\right)$ between two regions $C_{0}$ and $C_{1}$ (considering only two regions) is maximum (i.e., the point where the two classes are separated), we use following the equation:
  (2) $$\sigma_{B}^{2}\left(T\right)=\frac{{\left[m_{G}P_{1}\left(T\right)-m\left(T\right)\right]}^{2}}{P_{1}\left(T\right)\left[1-P_{1}\left(T\right)\right]}$$
  where, $m_{G}$ is the average gray level of the entire image and $P_{1}\left(T\right)$ is the occurrence probability into the region.
  To separate the blood vessels from the background, the general idea was to label each region of contiguous pixels with a different value, and with this value one can obtain the number of objects in the image which depends on the adjacency used.
3. Obtaining the skeletonized binary form
  Skeletonization of an image makes possible the classification, recognition and simplification of the objects within it, and one of its most important applications is that skeletonization reduces the structural form of an image to a graph. The skeleton tries to represent the shape of an object with a relatively small number of pixels and the position, orientation and length of the skeleton lines correspond to those equivalent to the original image.
  Once the vascular network is segmented we proceed to represent the image network with a relatively smaller number of pixels using the skeleton of the original image. This process generates a binary image which is stored in an array of $0^{\prime }s$ and $1^{\prime }s$, where the value of 1 corresponds to the image skeleton, while the value of 0 will be considered the image background.
  The region skeleton can be defined by the transform of the Median Axis Transformation (MAT) proposed by Blum et al. [19]. To define the MAT for each point p in R (the region), we seek if the point p is a close neighbor to B (edges of the region R).
  If *p* has more than one closed neighbor, it is said to belong to the median axis (i.e., it belongs to the skeleton) of R. It is important to notice that the concept of proximity depends on the definition of distance used. All the procedure is shown in Figure 1.
  It is worthwhile to mention that the skeletonized binary form is a 2D representation of the vascular network, this means that we only have 8 possible neighbors with respect to one single node. This apparently limitation can be overcome considering a 3D model and developing the same steps as those mentioned above. In recent years there have appear other models trying to resemble this process [20,21,22].

**Figure 2:**
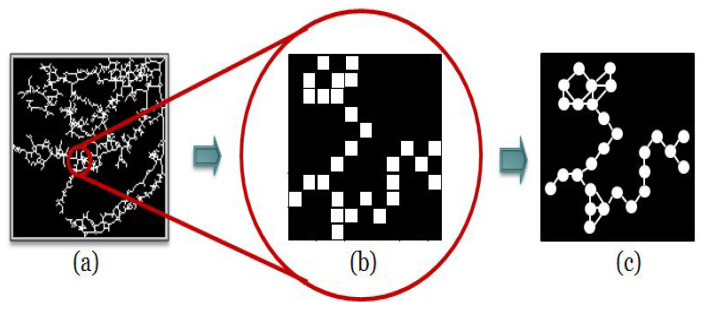
Modeling the complex network (**a**) binary skeletonized form. (**b**) Zoom of one part of the skeletonized binary form pixel by pixel (**c**) Network obtained after the assignation of nodes and edges to each pixel.

Once the skeletonized binary form has been obtained, we proceed to get the graph of the vascular network as follows: we postulate that every image pixel represents a single cell or point into a binary matrix. In order to form the network, it is proposed that every cell occupied (value of one) represents a node into the network. If any of these cells have other adjacent cells with value of one, i.e., occupied, it is possible that nodes are connected with their neighbors, so we assigned an edge between these two nodes, creating in this way the edges of the network, as shown in Figure 2.

### 2.2. Structure of the Network

We have built from a tomography image a complex network defined as a graph. With this in mind, we can measure some properties related to the complex network just created and try to understand the system behavior as a whole. Motivated by these ideas and considering that many biological networks share properties of the small world networks, we proceed to perform four measurements [17,23], namely,

- Clustering Coefficient: A common property of complex networks is the cliques that it forms. This inherent tendency to cluster is quantified by the clustering coefficient. Let us analyze briefly the concept; if we focus on a selected node *i* in the network, having $k_{i}$ edges which connect it to other $k_{i}$ nodes. If the nearest neighbours of the original node were part of a clique, there would be $k_{i}\left(k_{i}-1\right)/2$ edges between them. The ratio between the number $E_{i}$ of edges that actually exist between these $k_{i}$ nodes and the total number $k_{i}\left(k_{i}-1\right)/2$ gives the value of the node clustering coefficient *i*, as;
  $$C_{i}=\frac{2E_{i}}{k_{i}\left(k_{i}-1\right)}$$
  The clustering coefficient of the whole network is the average of all individual $C_{i}$.
- Degree Distribution: The way in which the degree of the nodes is distributed is characterized by the distribution function P(k), which is the probability that a randomly selected node has exactly *k* edges. For complex networks there are three types of important distributions, which determine different structures or topology of them, namely; Poisson Distribution, Exponential Distribution and Scale-Free Distribution.
  Networks that have a power-type distribution are called scale-free distributions or Power Law distributions. These networks arise in the context of network growth, in which each new node connects preferably to the nodes that are connected to the largest number of nodes in the network. Scale-free networks are also networks of the small world, because they have a coefficient of Clustering larger than a random network and the average of the shortest distance increases logarithmically with the number of nodes N, for this Distribution the probability density function is given by: $P\left(k\right)=Ck^{-\alpha }$ [1].
- Average path length: If we consider a unweighted graph *G* with the set of edges *E* and let $d(e_{1},e_{2})$, where $e_{1}$ and $e_{2}$, $e_{1},e_{2}\in E$ denote the shortest distance between $e_{1}$ and $e_{2}$. Then, the average path length $l_{G}$ is defined as;
  $$l_{G}=\frac{1}{n(n-1)}\sum_{i,j}d\left(e_{i},e_{j}\right)$$
  where *n* is the number of vertices of *G*.
- Fractal dimension: The fractal dimension is a statistical quantity that gives an indication of how completely a fractal appears to fill space, as one zooms down to finer scales. In order to obtain the fractal dimension we use the box counting method, this method of counting is used to determine the fractal dimension of an irregular object. It consists of covering the object with a grid and counting how many boxes of the grid contain parts of the object. This process is repeated, several times using boxes with sides equal to 1/2 of the size of the previous box [24]. The fractal dimension *d* is then the slope that is obtained from graphing LogN(r) vs Log(1/r) in an equivalent way, the negative of the gradient of graphing LogN(r) vs Log(r);
  $$d=\frac{\Delta logN(r)}{\Delta log(r)}$$

Taking into account the aforementioned properties, it is possible to perform these measurements over the binary skeletonized forms obtained from the images of four different patients. We report our results in Table 2.

**Figure 3:**
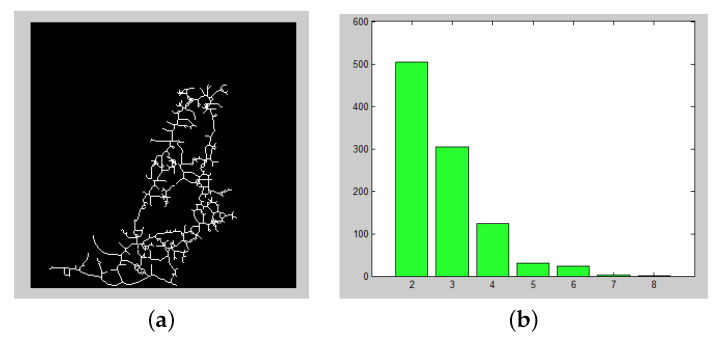
(**a**) Example of the Binary skeletonized form for patient B. (**b**) Degree distribution obtained from patient B.

Figure 3 depicts the skeletonized binary form and the degree distribution for the patient A, as an example of the network that we obtain for this special case.

### 2.3. Robustness Analysis

Angiogenesis is an important natural process that takes into account the growth of new blood vessels that occur in the body, both in healthy and ill hosts. Angiogenesis is now recognized as one of the critical events required for tumor progression [14]. In other words, cancerous growth is dependent on vascular induction and the development of new vascular supplies.

The idea of targeting angiogenesis to inhibit tumor growth was proposed more than three decades ago, and since then, several approaches to block or disrupt tumor angiogenesis have been explored. However, all of these have been focused on the understanding of the molecular behaviour, and only a few in other properties that are not apparent from the molecular point of view.

Recent studies suggest that a network’s connectivity pattern determines its robustness to external perturbations, such as removal of nodes or links [24]. To test this, we measured the effects of directed attacks and random failures on network organization. These measures were carried out as follows:

1. A given fraction of the vascular network nodes was eliminated from the original network. The nodes to be removed were either chosen as the most connected (directed attacks), or at random (random failures).
2. The network’s emerging was evaluated by calculating their structural properties, namely, the average path length, the clustering coefficient and the degree distribution.
3. The whole process was repeated for several fractions of removed nodes.

In Table 3 and Table 4 we showed the results of the robustness analysis for the real networks for the four patients taken from both directed (Table 3) and at random (Table 4) attacks.

Table 4 shows that for both patients (patient A and B), when we carried a direct attack, the statistical properties were lost after we eliminated nodes with five connections, i.e., the average path length become higher and the clustering coefficient lower, compared with the original. Our calculations also reveal that random removal nodes (see Table 3) have almost the same effect as in the direct attack when removing almost 15% from all the nodes.

## 3. Computational Modeling of Angiogenic Networks

### 3.1. Invasion Percolation Algorithm

Some years ago, Baish et al. [3] introduced an algorithm called Invasion Percolation in order to show that the fractal dimensions observed in tumor vasculature closely correspond to those produced by a statistical growth process known as Invasion Percolation [25]. In a more technical sense, Invasion Percolation is an algorithm that models the expansion of a network through a medium with randomly distributed heterogeneities. The resulting network always expands into the weakest available sites, yielding structures with voids on a wide range of length scales and pathways that are tortuous over many scales.

The Invasion-Percolation model is motivated by the problem of a fluid to be dispersed in a porous medium. This principle may be applied to any type of invasion process in which the path shows fluid passage resistance [25]. The porous medium may be represented as a network of pores which are connected between the pores. In an ideal medium the network can be viewed as a matrix in which the cells and their neighborhoods represent the pores and the connections between them. It is assigned random numbers to each cells in order to represent the pore size. The simulation of the fluid path through the pores consists of a series of discrete jumps, where each discrete step will be that offering less resistance (low random number). The Invasion-Percolation model involves a single time, in which the jump is generated in the matrix and provides a unique way to traverse the porous medium.

**Figure 4:**
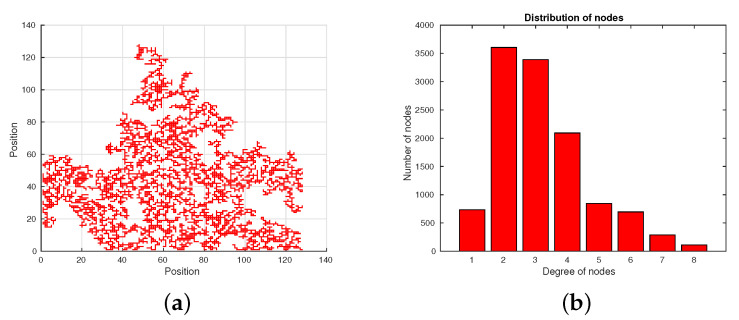
Example of the network generated by Invasion-Percolation algorithm. (**a**) Network generated by the algorithm. (**b**) Distribution of nodes generated by the same algorithm.

To show how the Invasion-Percolation algorithm works, we performed several experiments in which some networks were generated with this algorithm. Figure 4a depicts a single example of the synthetic network created by the Invasion-Percolation algorithm, for this case we have used a size of the matrix of 128 × 128 cells. Likewise, Figure 4b shows the distribution of nodes generated by the algorithm, in which it is observed how the distributions does not resembles to that obtained using the patient data (see for example Figure 3b for patient B).

The pseudo-code for Percolation is presented as follows:


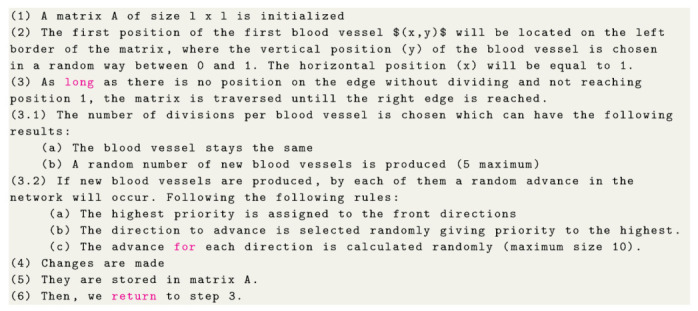


As was mentioned previously, the design of the Invasion Percolation algorithm intents to mimic or model the vasculature shown in real tumors. So, in order to have a trustworthy algorithm that could simulate angiogenic networks, we suggest that it is necessary that the Invasion Percolation algorithm could reproduce some of the properties shown in our angiogenic networks. For this purpose, all the synthetic networks produced by the Invasion Percolation were subjected to a structural analysis (i.e., measuring the average path length, the clustering coefficient and the degree of distribution) and a geometry analysis (i.e., measuring its fractal dimension), both previously proposed for studying real networks. The results obtained are presented in Table 5, which shows the study was made using synthetic networks. It is worthwhile to mention that in order to obtain Table 5 we have run our algorithm around five hundred times for different sizes of the matrix (32 × 32, 64 × 64, 128 × 128, 256 × 256 cells) to obtain as many as possible synthetic networks to work with.

A detailed analysis of Figure 4 and Table 5, it is shown that the Invasion-Percolation algorithm did not share the structural and geometrical properties shown by those obtained from real networks obtained in patients (see for instance, Table 2 and Figure 3). So, in our opinion the Invasion Percolation algorithm should be transformed in order to share the properties aforementioned. In order to address this point it was necessary to design a new algorithm in which we incorporated some strategies to better simulate not only the fractal properties shown by Baish [3], but also the structural properties revealed by the structural analysis made in real networks.

### 3.2. A New Algorithm Called Arga (Angiogenesis Random Growth Algorithm)

A new algorithm for the generation of angiogenic networks complying with the characteristics of structure and geometry of the modeled network obtained from tomographic images is proposed.

The new algorithm is motivated by the changes that could occur in the medium and responsible for the division of blood vessels. Its aim is to find a pathway through the medium, giving priority to the front feed (from left to right of the matrix) [25].

Similar to the Invasion-Percolation algorithm for network formation an ideal medium is assumed, i.e., it is homogeneous and symmetric. This medium will be represented by a matrix of size *n* x *n* and ideally when a change happened in the medium, this will be reflected in a change in the formation behaviour of the synthetic network. However, it is impossible to know these changes in situ. We will simulate these changes using a random variable which varies in time.

Basically, the pseudo-code for ARGA is presented as follows:


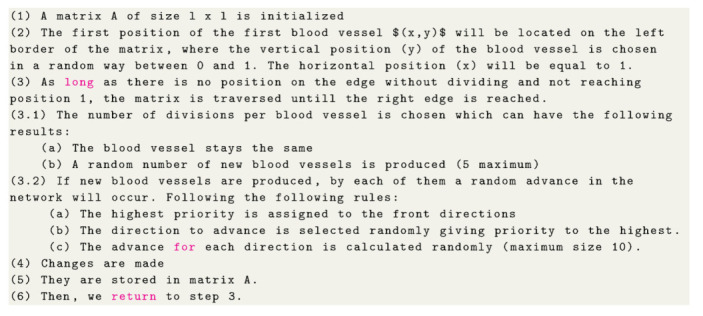


**Figure 5:**
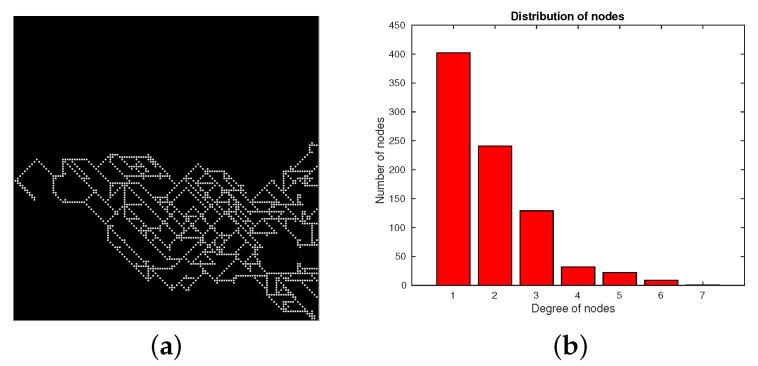
Example of a single network (with a size of 128 × 128 cells) generated by ARGA algorithm. (**a**) Network generated by the algorithm. (**b**) Distribution of nodes generated by the same algorithm

To show how the ARGA algorithm works, we performed several experiments in which some networks were generated with this algorithm. Figure 5a depicts a single example of the synthetic network created by the ARGA algorithm, for this case we have used a size of the matrix of 128 × 128 cells. Likewise, Figure 5b shows the distribution of nodes generated by the algorithm, in which it is observed how the distributions resembles in a better way to those obtained using the patient data (see for example Figure 3b for patient B).

Once we have our algorithm running, we developed hundreds of simulations (it is worthwhile to mention that in order to obtain Table 6 we have run our algorithm around five hundred times for different sizes of the matrix (32 × 32, 64 × 64, 128 × 128, 256 × 256 cells)) to obtain as many as possible synthetic networks to work with. All these synthetic networks produced by ARGA were subjected to a structural analysis (i.e., measuring the average path length, the clustering coefficient and the degree of distribution) and a geometry analysis (i.e., measuring its fractal dimension), both previously proposed for studying real networks. The results obtained are presented in Table 6, using these synthetic networks, the table only shows the average values and the standard deviation obtained for all the synthetic networks.

### 3.3. Robustness Analysis for the Arga Algorithm

Below are the results obtained from the robustness analysis made to the synthetic networks produced by the ARGA algorithm, using the previous cuts of size (32 × 32, 64 × 64, 128 × 128 and 256 × 256 cells). As explained in Section 2.3 (for the real networks case), the robustness analysis was carried out by attacking the networks, randomly and in directed way, and then characterized them using the proposed structure and geometry analyzes. The full results of the robustness analysis for the case of the networks created by the ARGA algorithm can be seen in Table 7 and Table 8. In these we have shown the results of the robustness analysis for the synthetic networks for four different sizes taken from both directed (Table 7) and random (Table 8) attacks.

## 4. Concluding Remarks

An analysis has been carried out on the vascular angiogenic network on four patients with Hepato-Cellular Carcinoma (HCC). This analysis consisted of measuring a number of statistical properties of a vascular network obtained from four digital tomographies and digitalized until a binary skeletonized scheme is obtained. From this, we generated a network of nodes and edges which represent the original angiogenetic vascular network. Some interesting observations arising from these measurements are:

- The clustering coefficient in all 16 generated networks is less than 0.4. This indicates that they were well connected networks.
- The degree distribution in all the networks have an exponential tail with the distribution exponent, between 0.6 and 1.1.
- The average path length is small in all the networks being between 0.009 and 0.071.
- The fractal dimension is found to be around 1.4.

Many authors have considered that: “*Tumor consists of a chaotic, poorly organized vasculature, with tortuous, irregularly shape, and leaky vessels that are often unable to support efficient blood flow and leading to an aberrant vascular system*”. From our observations the above consideration is incorrect because we have shown that there is a well connected network (high clustering coefficient); besides, the network has an efficient communication. This is reflected in a small average path length. So, when observing in situ a poorly organized shape, it does not take into account that there are good structural properties that offer support for various dynamical processes, i.e., it is thought that the network topology plays a crucial role, which supports an efficient blood flow among other dynamical properties.

The high interest in scale-free networks in literature might give the impression that all complex networks in nature have power-law degree distributions. It is true for several complex networks of highest interest in the scientific community, such as the World Wide Web, social networks among others, that in all of them the degree distribution has a power-law tail. However, some other networks such as neural and power grid showed exponential degree distributions in literature, and these are called evolving networks [3]. In our case, we have exponential distributions for all the vascular angiogenic networks generated. This means having and evolving a network with aging effects and growth constraints that leads to this exponential decay.

A study of the robustness of the generated angiogenic vascular network has been carried out. We studied the robustness of the network analyzing the connectivity pattern subjected to external perturbations, such as the removal of nodes or links. To test this, the effects of directed attacks and random failures on the network organization were measured.

This study shows that both patients (patient A and B), when we carried a direct attack, their statistical properties were lost after we eliminated nodes with five connections, i.e., the average path length become higher and the clustering coefficient lower, compared with the original. Our calculations also reveal that random removal nodes have almost the same effect as in the direct attack when removing almost 15% from all the nodes.

Taking into account a new kind of algorithm (ARGA) it was shown that this algorithm simulates in a better way the growth of the vascular network than the Invasion-Percolation algorithm. This is clear because with this algorithm it was possible to reproduce, in a better way, the structural and geometric measurements for the real network than with the Invasion-Percolation algorithm. Some interesting observations arising from these measurements (see Table 6) are:

- The clustering coefficient in all the generated networks is less than 0.3. This indicate that they were well connected networks, as in the case of real data.
- The degree distribution in all the networks have an exponential tail with the distribution exponent, between 2.9 and 3.78.
- The average path length is small in all the synthetic networks being between 0.03 and 0.09, as in the case of real data.
- The fractal dimension is found to be around 1.4, as in the case of real data.

Furthermore, both algorithms were subjected to a variety of direct attacks and random failures and, for the ARGA algorithm the same effect as in the patients A and B was observed, i.e., all the statistical properties were lost in the same manner as the real networks (see Table 7 and Table 8). However, the Invasion Percolation did not share the behaviour (the experiments are not included in this article) of real networks as the ARGA algorithm did for the case of direct and random attacks. This indicates that in order to simulate in a better way real networks the use of the ARGA algorithm will produce synthetic networks more similar to the real ones.

Taking into account the considerations of the last two paragraphs, and based on the statistical properties of the network, some possible therapies could be suggested. We think that more clinical research related to the structure and robustness of the vascular network for the angiogenic process could be done in order to prove the latter hypothesis.

## Figures and Tables

**Table 1:** Characteristics of the four patients studied in the INNSZ, all with Hepato-Cellular Carcinoma (HCC).

Patient	Sex	Age	Date
A	Female	44	23 November 2007
B	Male	57	5 February 2008
C	Female	63	30 October 2007
D	Female	55	8 March 2007

**Table 2:** General structural properties of the four networks. For each network we have indicated the number of nodes (size), the average degree *k*, the clustering coefficient *C*, the average path length *l*, the fractal dimension *D* and the distribution exponent α (this exponent was calculated taking into account a power law distribution).

Image	Size	k	C	l	D	α
A (32 × 32)	98	2	0.169	0.061	1.304	3.256
A (64 × 64)	340	3	0.279	0.035	1.395	3.034
A (128 × 128)	630	2	0.231	0.020	1.357	4.0624
A (256 × 256)	2301	2	0.226	0.010	1.409	4.334
B (32 × 32)	79	2	0.122	0.066	1.278	3.302
B (64 × 64)	234	2	0.201	0.036	1.332	4.168
B (128 × 128)	1248	2	0.180	0.009	1.470	4.481
B (256 × 256)	2247	2	0.187	0.009	1.396	4.222
C (32 × 32)	111	2	0.156	0.063	1.342	2.552
C (64 × 64)	211	2	0.152	0.029	1.301	3.507
C (128 × 128)	987	2	0.214	0.015	1.425	2.703
C (256 × 256)	3570	2	0.230	0.009	1.494	2.907
D (32 × 32)	103	2	0.251	0.071	1.322	3.101
D (64 × 64)	428	2	0.207	0.026	1.463	3.320
D (128 × 128)	894	2	0.204	0.014	1.390	3.921
D (256 × 256)	1260	2	0.169	0.010	1.291	4.250

**Table 3:** Robustness analysis for two networks using a random attack. For each network we have indicated the number of nodes (*N*), and the number of nodes eliminated randomly (EN), beginning with 1% of the nodes (corresponding to the first row of each patient). It is worthwhile to note that 1% corresponds, in the first case, to 3 disconnected nodes; however, when we disconnect these 3 nodes other adjacent nodes are also disconnected, giving 6 in total disconnected nodes. We did the same for 5%, 10% and finally 15%, the average degree *k*, the average length *l*, the clustering coefficient *C*, the fractal dimension *D* and the exponent of the distribution α. In all the attacks we used images of 64 by 64 pixels.

PATIENT A	*N*	EN	*k*	*l*	*C*	*D*	α
	340	6	3	0.0339	0.279	1.390	0.740
	340	18	2	0.039	0.262	1.387	0.687
	340	35	2	0.051	0.267	1.326	0.704
	340	52	3	0.122	0.303	1.144	0.550
**PATIENT B**	N	EN	k	l	C	D	α
	234	3	2	0.045	0.188	1.396	0.944
	234	13	2	0.036	0.187	1.272	1.074
	234	24	2	0.077	0.227	1.083	0.626
	234	36	2	0.218	0.254	1	0.548

**Table 4:** Robustness analysis for two networks using a direct attack. For each network we have indicated the number of nodes (*N*), and the number of nodes eliminated (EN), beginning with the nodes with 7 connections (this corresponds to the first row of each patient and in parenthesis are the remaining nodes), then the nodes with 6 (corresponding to the second row) and so on, the average degree *k*, the average length *l*, the clustering coefficient *C*, the fractal dimension *D* and the exponent of the distribution α. In all the attacks we used images of 64 by 64 pixels.

PATIENT A	*N*	EN	*k*	*l*	*C*	*D*	α
	340	6 (334)	2	0.03	0.262	1.392	0.7984
	340	19 (315)	2	0.036	0.217	1.391	1.0072
	340	21 (294)	2	0.048	0.226	1.237	0.78551
	340	64 (230)	2	0.060	0.118	1.089	1.292
**PATIENT B**	N	EN	k	l	C	D	α
	234	1	2	0.036	0.197	1.332	1.008
	234	1	2	0.036	0.198	1.332	1.223
	234	7	2	0.0364	0.164	1.326	0.870
	234	28	3	0.110	0.144	1	0.778

**Table 5:** General structural properties for networks created by the Invasion-Percolation algorithm for different matrix sizes. After several simulations (we only have reported the average values for each measure) for each size, we have indicated the average number of nodes (average size) *N*, the average degree *k*, the average clustering coefficient *C*, the average path length *l* and the average of the fractal dimension *D*, also we have added the standard deviation (in parenthesis) for each size and for each measure.

Matrix	N	Z	C	l	D
32 × 32	310 (79.14)	4	0.49 (0.02)	0.007 (0.008)	1.64 (0.092)
64 × 64	837 (235.3)	4	0.44 (0.14)	0.08 (0.014)	1.62 (0.07)
128 × 128	4373 (1515.95)	4	0.477 (0.007)	0.018 (0.002)	1.72 (0.07)
256 × 256	16441 (5876)	4	0.47 (0.006)	0.009 (0.0019)	1.75 (0.075)

**Table 6:** General structural properties for networks created by the ARGA algorithm for different matrix sizes. For each size we have indicated the average number of nodes (average size) *N*, the average degree *k*, the average clustering coefficient *C*, the average path length *l*, the average of the fractal dimension *D* and the average exponent of the distribution α (this exponent was calculated taking into account a Poisson distribution), also we have added the standard deviation (in parenthesis) for each size and for each measure.

Matrix	N	Z	C	l	D	α
32 × 32	110 (16.2)	3	0.26 (0.08)	0.032 (0.001)	1.32 (0.125)	2.92 (0.67)
64 × 64	458 (174.8)	3	0.27 (0.04)	0.03 (0.004)	1.48 (0.09)	3.094 (0.51)
128 × 128	1772 (490.31)	3	0.27 (0.025)	0.08 (0.002)	1.56 (0.06)	3.8 (0.48)
256 × 256	8522 (3961)	3	0.295 (0.02)	0.09 (0.001)	1.6 (0.08)	3.78 (0.61)

**Table 7:** Robustness analysis for synthetic networks using a random attack. For each network we have indicated the number of nodes (*N*), the number of nodes eliminated randomly (EN) beginning with 1% of the nodes (corresponding to the first row to the size of the network), for 5%, 10% and finally 15% respectively, the average degree *k*, the average length *l*, the clustering coefficient *C* and the fractal dimension *D*.

Size (32 × 32 cells)	*N*	EN	*k*	*l*	*C*	*D*
1%	108	1	3	0.0839	0.3754	1.4235
5%	108	7	2	0.0819	0.3870	1.415
10%	108	12	2	0.0737	0.3606	1.3853
15%	108	18	3	0.1030	0.3545	1.2682
**Size (64 × 64 cells)**	N	EN	k	l	C	D
1%	483	6	3	0.0412	0.3345	1.4860
5%	483	25	3	0.0409	0.3352	1.402
10%	483	49	3	0.0456	0.3185	1.450
15%	483	73	3	0.346	0.3580	1.340
**Size (128 × 128 cells)**	N	EN	k	l	C	D
1%	1463	32	2	0.017	0.2708	1.5544
5%	1463	837	2	0.026	0.2767	1.3553
10%	1463	911	2	0.0298	0.2668	1.378
15%	1463	1216	2	0.0319	0.2780	1.19
**Size (256 × 256 cells)**	N	EN	k	l	C	D
1%	4231	1904	2	0.009	0.2443	1.4980
5%	4231	617	2	0.0094	0.2592	1.5412
10%	4231	2060	2	0.0064	0.2757	1.4808
15%	4231	3767	2	0.011	0.2732	1.200

**Table 8:** Robustness analysis for the synthetic networks using a direct attack. For each network we have indicated the number of nodes (*N*), the number of nodes eliminated (EN) beginning with the nodes with 7 connections (this corresponds to the first row), then the nodes with 6 (corresponding to the second row) and so on, the average degree *k*, the average length *l*, the clustering coefficient *C* and the fractal dimension *D*.

Size (32 × 32 cells)	*N*	EN	*k*	*l*	*C*	*D*
7	108	0	3	0.0839	0.3754	1.4253
6	108	7	2	0.07020	0.2799	1.409
5	108	6	2	0.0807	0.313	1.411
4	108	21	2	0.0758	0.1760	1.557
**Size (64 × 64 cells)**	N	EN	k	l	C	D
7	483	5	3	0.0411	0.3248	1.4867
6	483	26	2	0.03834	0.3016	1.4763
5	483	48	2	0.0334	0.2709	1.4685
4	483	126	2	0.1023	0.1031	1.05
**Size (128 × 128 cells)**	N	EN	k	l	C	D
7	1463	4	2	0.0180	0.2614	1.5592
6	1463	21	2	0.0177	0.2472	1.5577
5	1463	59	2	0.0168	0.2310	1.5540
4	1463	1354	2	0.0356	0.068	1.1899
**Size (256 × 256 cells)**	N	EN	k	l	C	D
7	4231	14	2	0.0105	0.2591	1.5716
6	4231	96	2	0.0102	0.2439	1.5697
5	4231	329	2	0.0093	0.2260	1.5610
4	4231	3971	2	0.022	0.2239	1.03
